# Methylprednisolone pulse therapy and intravenous cyclophosphamide therapy combined with cocktail therapy in severe pediatric Henoch-Schönlein purpura nephritis patient

**DOI:** 10.1007/s13730-012-0056-8

**Published:** 2013-01-12

**Authors:** Hiroaki Kanai, Anna Kobayashi, Kyoko Matsushita, Emi Sawanobori, Kanji Sugita, Kosuke Higashida

**Affiliations:** Department of Pediatrics, Faculty of Medicine, University of Yamanashi, Shimokato 1110, Chuo-city, Yamanashi 409-3898 Japan

**Keywords:** Henoch-Schönlein purpura nephritis, Intravenous cyclophosphamide therapy, Methylprednisolone pulse therapy, Nephrotic syndrome

## Abstract

Henoch-Schönlein purpura (HSP) is a common self-limited vasculitis in children. The long-term prognosis depends on renal involvement. In severe Henoch-Schönlein purpura nephritis (HSPN) patients, >50 % have crescent formation and nephrotic syndrome that are important predicted outcomes. Therefore, for such patients, an aggressive immunosuppressive therapy is needed to prevent the progression. However, there is no consensus for an appropriate therapeutic regimen for severe pediatric HSPN patients. In this paper, we have reported on a 6-year-old boy who presented with HSPN with nephrotic syndrome and severe histopathological abnormalities; he was diagnosed with International Study of Kidney Disease in Children (ISKDC) grade IVb. Despite treatment with methylprednisolone pulse therapy, followed by oral prednisolone and dipyridamole; the nephrotic syndrome persisted. Subsequently, intravenous cyclophosphamide therapy (IVCY) (500–1,000 mg m^−2^ once a month for 7 months; total 6,000 mg m^−2^) was administered, followed by azathioprine and enarapril. Within 7 months of disease onset, complete remission was achieved. After 22 months of the initial renal biopsy, the second biopsy was performed to confirm treatment efficacy. Histopathological findings improved, and ISKDC grade IIIa was diagnosed. Even after 5 years of HSPN onset, complete remission and normal renal function is maintained. Although our evidence is restricted to single patient, we have shown that MPT and IVCY combined with cocktail therapy may be an effective treatment for severe pediatric HSPN.

## Introduction

Henoch-Schönlein purpura nephritis (HSPN) occurs in 20–60 % of Henoch-Schönlein purpura (HSP) patients. Renal involvement mainly causes the morbidity and mortality. The majority of the children with HSPN present only hematuria and/or low-grade proteinuria, and have a good chance to recover with favorable renal prognosis. However, patients with >50 % crescent formation, nephrotic syndrome, or renal insufficiency at onset are at a risk of poor outcome [1]. Previous studies have reported severe pediatric HSPN patients treated with immunosuppressive agents, including corticosteroid, cyclophosphamide (CYC), cyclosporin A (CsA), and azathioprine (AZA), but the data from these interventions are not conclusive, as discussed in the recent Cochrane review [2].

Further, it was recently reported that a patient with severe HSPN was successfully treated by intravenous cyclophosphamide therapy (IVCY) combined with cocktail therapy, proposing a new type of treatment for this subset of severe HSPN patients [3–5]. We report a case of 6-year-old boy with HSPN who presented with nephrotic syndrome and severe histopathological features (>50 % crescent formation). He was successfully treated with methylprednisolone pulse therapy (MPT) and IVCY followed by cocktail therapy (prednisolone, AZA, dipyridamole, and enarapril).

## Case report

A 6-year-old boy was admitted to our hospital 2 weeks after HSPN onset, because proteinuria and hematuria had worsened. His family and medical history were unremarkable, and purpura, abdominal pain, or arthritis was not evident on physical examination. His blood pressure was 98/65 mmHg; serum total protein level 4.5 g dl^−1^, albumin 2.2 g dl^−1^, blood urea nitrogen 8.0 mg dl^−1^, serum creatinine 0.38 mg dl^−1^, estimated glomerular filtration rate (eGFR) by the Schwartz formula [6] 111 ml min^−1^ per 1.73 m^2^, IgA 138 mg dl^−1^. Dipstick urinalysis showed results of 3+ for occult blood and 3+ for protein. The early morning urine-protein to creatinine ratio was 19.2 g/g Cr.

**Fig. 1:**
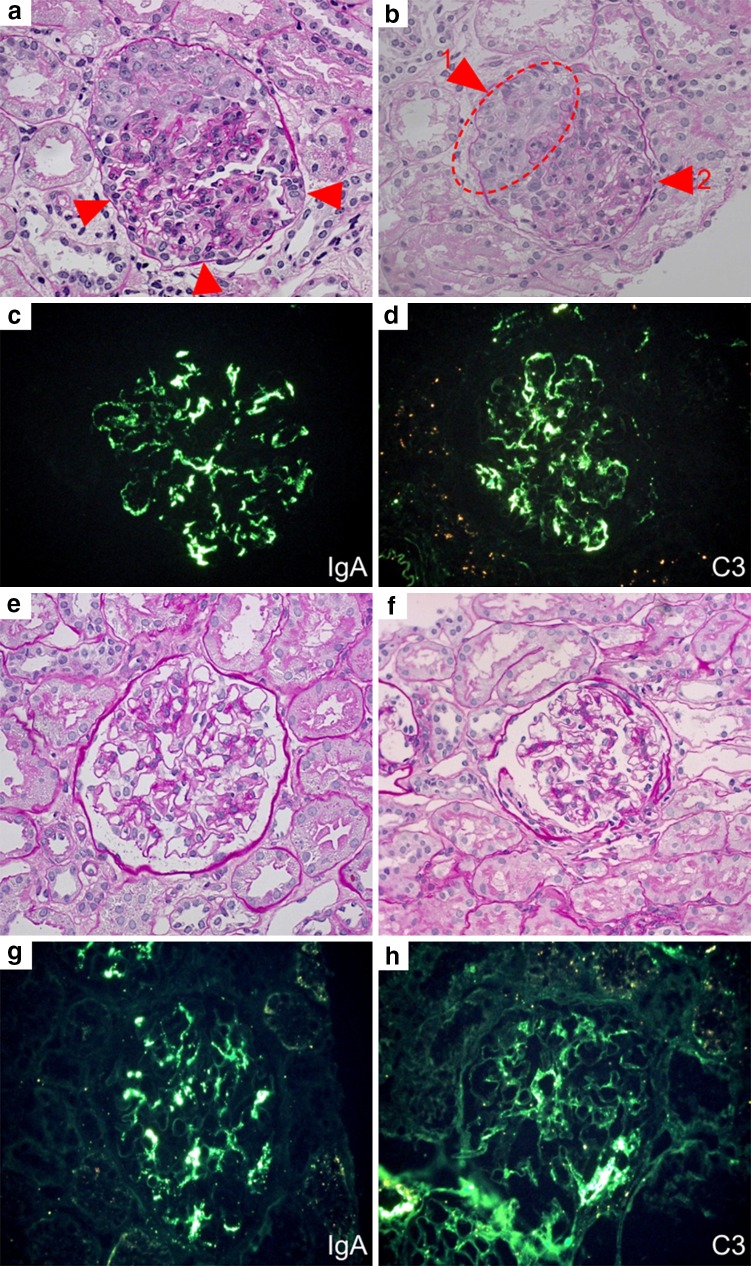
Renal biopsy findings. Panels **a**–**d** Initial renal biopsy findings **a** Light microscopy reveals diffuse global mesangial proliferation, and infiltration of polymorphonuclear leukocytes (*arrow*). Periodic acid-Schiff (PAS) staining, original magnification, ×200. **b** Cellular crescent (*arrow 1*), and adhesion (*arrow 2*). PAS staining, original magnification, ×200. **c**, **d** Immunofluorescence (IF) analysis of IgA and C3 showing predominant granular deposition in the mesangial area; original magnification, ×200. Panels **e**–**h** Second renal biopsy findings **e**, **f** Light microscopy revealed that the mesangial proliferation was extremely improved. PAS staining; original magnification, ×200. **g**, **h** IF analysis of IgA and C3, showing predominant granular deposition in the mesangial area, similar to the initial biopsy findings. However, the intensity is reduced; original magnification, ×200

On initial biopsy, light microscopy revealed 29 glomeruli, diffuse mesangial proliferation, infiltration of polymorphonuclear leukocytes in glomerular capillaries, and presence of cellular crescents (16 of 29 glomeruli, 55 %) and adhesions (6 of 29 glomeruli, 21 %). Glomerulosclerosis and interstitial lesions were absent. Immunofluorescence (IF) showed predominant granular deposition of IgA (3+), C3 (3+), IgG (+), and IgM (+) in the mesangial areas (Fig. 1). Based on the histopathological findings, he was diagnosed with International Study of Kidney Disease in Children (ISKDC) grade IVb.

**Fig. 2:**
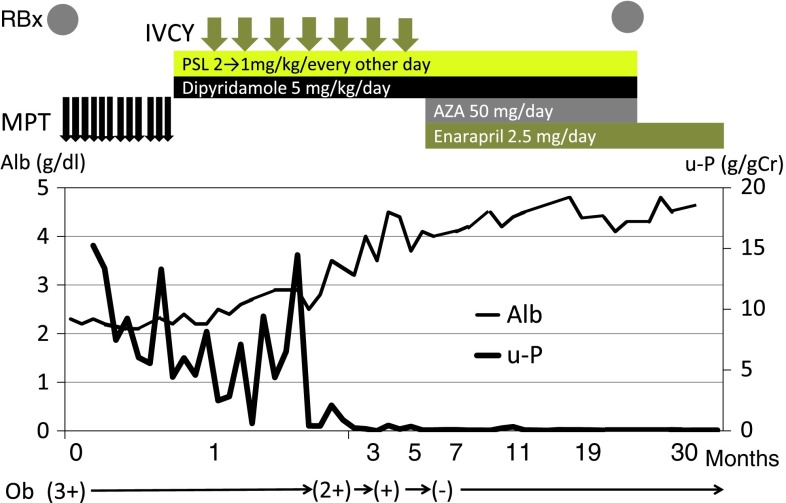
Patient’s clinical course. After initial renal biopsy, treatment with MPT, followed by cocktail therapy (oral prednisolone, and dipyridamole) was unsuccessful, after which he was treated by IVCY therapy followed by azathioprine and enarapril. After this, proteinuria and hematuria gradually improved and the patient achieved complete remission within 7 months of the onset of HSPN. *MPT* methylprednisolone pulse therapy, *IVCY* intravenous cyclophosphamide therapy, *PSL* prednisolone, *AZA* azathioprine, *Ob* occult blood, *RBx* renal biopsy

After obtaining written informed consent, the patient first received MPT (30 mg kg^−1^ day^−1^ for three consecutive days/week) for 4 weeks, followed by cocktail therapy comprising oral prednisolone (2 mg kg^−1^ day^−1^) every other day, and dipyridamole (5 mg kg^−1^ day^−1^). The patient was sequentially treated with IVCY (500–1,000 mg m^−2^ once a month for 7 months; total 6,000 mg m^−2^), followed by AZA (50 mg day^−1^) and enarapril (2.5 mg day^−1^) After 3 months, the dosage of prednisolone was decreased every alternate day by 1 mg kg^−1^ day^−1^. Post MPT plus IVCY, both proteinuria and hematuria gradually improved. The proteinuria resolved within 2 months of initiation of IVCY treatment, and hematuria, within 4 months (Fig. 2). Tonsillectomy was then performed as an add-on therapy 7 months after initiation of IVCY treatment in order to maintain remission.

The second renal biopsy was performed 22 months after the first biopsy to confirm treatment efficacy. Physical examination did not reveal any remarkable findings. Blood pressure and biochemical test results were normal: serum total protein 6.4 g dl^−1^, albumin 4.4 g dl^−1^, blood urea nitrogen 11.2 mg dl^−1^, serum creatinine 0.42 mg dl^−1^, eGFR 112 ml min^−1^ per 1.73 m^2^, cystatin C 0.89 mg l^−1^, and IgA 68 mg dl^−1^. Urinalysis was normal. Light microscopy revealed 54 glomeruli, focal segmental mesangial proliferation, and presence of adhesions (7 of 54 glomeruli, 13 %). Glomerulosclerosis, crescent formation, and interstitial lesions were absent. The IF showed predominant granular deposition of IgA (2+), C3 (+), IgG (+), and IgM (+) in the mesangial areas; however, with less intensity (Fig. 1). Based on the histopathological findings, ISKDC grade IIIa was diagnosed.

Medication except enarapril was tapered off 24 months after HSPN onset (Fig. 2). Within 5 years of onset, blood pressure, urinary findings, and renal function were normal (serum creatinine, 0.67 mg dl^−1^ and cystatin C, 0.67 mg l^−1^).

## Discussion

CYC is used in pediatric renal diseases primarily for rapidly progressive glomerulonephritis, lupus nephritis, and nephrotic syndrome. Despite uncontrolled study, early treatment with oral PSL and CYC (2 mg/kg/day for 8 weeks) was found to be effective in nine children with clinically severe forms of HSPN. At the latest follow-up (mean interval 78 months), all except two showed negative proteinuria while no patient showed renal impairment [7]. However, randomized study by Tarshish et al. [8] showed that oral CYC (90 mg/m^2^/day for 42 days) as single therapy was less effective than supportive therapy for histopathologically severe HSPN patients. At final follow-up (mean time, 3.71 years), 3 out of 28 patients (11.0 %) in the oral CYC group and 4 out of 28 patients (14.3 %) in the control group progressed to end-stage renal disease. No differences in outcome were found between the treatment trial and the control group. On the other hand, Rasche et al. [9] prospectively reported that IVCY (750 mg m^−2^ once a month for 6 months) and low dose oral prednisolone is effective in decreasing proteinuria and preserving renal function in patients with advanced progressive IgA nephropathy, which is similar to HSPN. The loss of renal function per year significantly reduced from 16 % before therapy to 4 % after therapy. Another prospective study by Tumlin et al. [10] has shown that IVCY (500–750 mg m^−2^ once a month for 6 months) combined with MPT reduced proliferative lesions and proteinuria, and stabilized renal function in patients with the crescentic IgA nephropathy. To determine the long-term efficacy of the treatment, 12 treated patients were compared to 12 historical controls matched for the severity of IgA on initial biopsy. After 36 months, the incidence of end-stage renal disease in the treated group was lower (1/12) than that in the historical controls (5/12). Furthermore, in two recent reports, IVCY was effective in treating adults with HSPN who were resistant to various treatments [3, 5]. López Meiller et al., used 1000 mg m^−2^ once a month for 6 months, and Namba et al., used 375 mg m^−2^ once a month for 5 months to treat HSPN in adults. In addition, there is another report that showed the efficacy of IVCY (500–750 mg m^−2^ once a month for 1–6 months) combined with corticosteroids, and angiotensin-converting enzyme inhibitor/angiotensin receptor blocker for pediatric patients with severe HSPN and IgA nephropathy [4]. Further, the meta-analysis by Groot et al. [11] demonstrated that in patients with Wegener’s granulomatosis and microscopic polyangiitis, IVCY is more effective in preventing relapses and has significantly lower risk of infections and leucopenia than oral CYC. For these reasons, we judged that, although the evidence about the long-term renal prognosis of IVCY is insufficient, IVCY was effective for achieving prompt complete remission and maintaining the remission. Therefore, we selected a similar IVCY treatment regimen (500–1,000 mg m^−2^ once a month for 7 months; total 6,000 mg m^−2^) and not the oral CYC treatment regimen.

In our patient, histopathology indicated disease of grade ISKDC IVb and he developed nephrotic syndrome at the onset; so it was predicted that renal prognosis was very poor. Few papers have reported about the long-term renal prognosis of severe HSPN patient treated with various immunosuppressive drugs. We referred to various immunosuppressive treatment regimens except CYC reported previously. Niaudet et al. [12] reported that MPT followed by oral prednisolone (3.5 months) was effective for severe HSPN, but about 10 % of patients in their study progressed to end-stage renal disease at the latest follow-up, 1–16 years after the initiation of therapy. Dual therapy with AZA and oral steroids resulted in similar outcomes at follow-up after 32 months [13]. In addition, Shenoy et al. [14] have reported the efficacy of treatment protocol comprising daily steroids and oral CYC (2–2.5 mg/kg/day for 8–12 weeks) followed by AZA. After a mean follow-up period of 7 years following presentation, 14.8 % had progressed to end-stage kidney failure. From the above-mentioned reports, we judged that the effect of MPT and cocktail therapy (steroid, AZA, and oral CYC) are insufficient for our patient. Therefore, it was necessary for further immunosuppressive therapies to be provided as well as IVCY for maintaining complete remission and preserving renal function.

Our patient underwent tonsillectomy as an add-on therapy when there was no urinary abnormality. In the recent reports, tonsillectomy was associated with a favorable renal outcome of IgA nephropathy in terms of clinical remission and delayed renal deterioration even in non-steroid-treated patients [15]. It was also reported that tonsillectomy and MPT compared with MPT alone has a favorable effect on long-term renal survival in patients with IgA nephropathy [16]. There have been some reports on the efficacy of combination of tonsillectomy and MPT in treating severe HSPN in adults and children [17–19]. It was concluded that tonsillectomy appeared to contribute to the prevention of HSPN relapses and the maintenance of the renal function. In another study, Inoue et al. reported that tonsillectomy alone might be able to prevent the complications of HSPN, thereby contributing to early recovery from HSP in children [20]. It is possible that tonsillectomy for HSPN may alter renal progression. However, there are no studies with discussion about whether monotherapeutic tonsillectomy is effective or not for long-term renal prognosis for HSPN, and even IgAN, when urinary findings are normal. Hence, further studies about the efficacy of monotherapeutic tonsillectomy for severe HSPN patients without urinary abnormality are needed.

Further, CsA therapy is performed in combination or as an add-on therapy with corticosteroids. There have been a few case reports suggesting that CsA may have a beneficial effect in children with severe HSPN [21–23]. However, the effect is restrictive, and the optimal dose, target trough level concentration, and duration of CsA treatment are not known in the treatment of HSPN. Furthermore, side effects of CsA were reported in 82 % of cases [21]. The most common side effects of CsA were hirsutism (82 %), gingival hypertrophy (45 %), and transient elevation in serum creatinine (45 %). The CsA nephrotoxicity for HSPN, which is characterized by arteriolopathy and striped interstitial fibrosis with tubular atrophy, is not well studied yet.

The CYC is known to cause oncogenicity and gonadal dysfunction, which is influenced by cumulative dose, sex, and age. Gonadal dysfunction affects boys more than girls, and in the puberty stage than in prepuberty. According to a meta-analysis of renal disease and malignant tumor in prepubertal boys, the minimum cumulative dose causing gonadal dysfunction is 300–400 mg kg^−1^ [24]. Our patient was prepubertal and the cumulative dose he received was 6,000 mg m^−2^ (approximately 200 mg kg^−1^), which can be considered safe. However, it is recently reported that gonadal dysfunction can occur even when less than 300–400 mg/kg of CYC is administered [25]. Therefore, careful informed consent is necessary. Nevertheless, malignancy is rare, with only one report of association between malignancy and CYC in a 6-year-old boy with nephrotic syndrome who developed bladder cancer after 4 years of treatment with 49.5 g CYC [26]. In addition, combination of enough hydration and administration of mesna is established in preventing hemorrhagic cystitis. Therefore, our IVCY treatment regimen can be regarded as safe.

The importance and efficacy of a prompt treatment has been well demonstrated in achieving complete remission in patients with severe HSPN [7, 27, 28]. All these reports suggest that the early suppression of disease activity by immunosuppressant or elimination of circulating immune complexes is essential for attaining clinical remission of HSPN. However, even after such aggressive and prompt therapeutic approaches, a subset of HSPN patients do not respond well and may progress to end-stage renal failure through successive flare-ups [27–29]. Therefore, it was necessary that our patient achieved remission promptly. We observed that only MPT was not effective in our patient and hence, treated him with IVCY combined with cocktail therapy (PSL, AZA, dipyridamole, and ACE inhibitor). As a result, he achieved complete remission promptly, and on the second biopsy, the histopathological findings had significantly improved (crescents had disappeared). After 5 years of the onset of HSPN, complete remission and normal renal function are maintained only by ACE inhibitor. However, renal impairment sometimes occurs among patients with transient resolution of renal manifestations, especially for histologically severe HSPN. Therefore, careful follow-up is necessary for patients with HSPN.

In conclusion, our case demonstrated that MPT plus IVCY treatment combined with cocktail therapy could not only achieve early complete remission but also maintained remission and normal renal function. Therefore, it appears that IVCY combined with cocktail therapy as add-on therapy to MPT is effective in treating severe HSPN, which could not be promptly treated with other agents. Our case provides a theoretical ground for the use of IVCY in the treatment of severe HSPN, together with an anecdotal evidence of its efficacy. However, further clinical trials are necessary to confirm whether IVCY is effective in treating severe HSPN.
